# Particularities of R134a Refrigerant Temperature Variations in a Transient Convective Regime during Vaporization in Rectangular Microchannels

**DOI:** 10.3390/mi13050767

**Published:** 2022-05-13

**Authors:** Ioan Mihai, Cornel Suciu, Claudiu Marian Picus

**Affiliations:** 1 Faculty of Mechanical Engineering, Automotive and Robotics, Stefan cel Mare University, 720229 Suceava, Romania; claudiu.picus@usm.ro; 2 Faculty of Electrical Engineering and Computer Science, Stefan cel Mare University, 720229 Suceava, Romania

**Keywords:** microchannels, R134a refrigerant, vaporization, heat exchanger

## Abstract

An analysis of the R134a (tetrafluoroetane) coolant’s non-stationary behavior in rectangular microchannels was conducted with the help of a newly proposed miniature refrigerating machine of our own design and construction. The experimental device incorporated, on the same plate, a condenser, a lamination tube and a vaporizer, all of which integrated rectangular microchannels. The size of the rectangular microchannels was determined by laser profilometry. R-134a coolant vapors were pressurized using a small ASPEN rotary compressor. Using the variable soft spheres (VSS) model, the mean free path, Knudsen and Reynolds numbers, as well as the dimensionless velocity profile can be assessed analytically. In order to determine the average dimensionless temperature drop in the vaporizer’s rectangular microchannels, in non-stationary regime, an analytical solution for incompressible flow with slip at the walls, fully developed flow and laminar regime was used, by aid of an integral transform approach. In the experimental study, the transitional distribution of temperature was tracked while modifying the R134a flow through the rectangular microchannels. Coolant flow was then maintained at a constant, while the amount of heat absorbed by the vaporizer was varied using multiple electric resistors. A comparative analysis of the analytical and experimental values was conducted.

## 1. Introduction

The increasingly advanced miniaturization of electronic components and their exposure to increasingly higher voltages has resulted in the development of high-performance cooling solutions. In practice, the aim is to decrease the size of these cooling systems, whilst at the same time try to conserve their efficiency. Although not limited to these fields, the following applications can be mentioned: microelectronics (first introduced by Tuckerman and Pease) [1,2,3,4,5]; micro-electro-mechanical systems (MEMS) [6,7,8]; micro-heat pipe spreaders [4]; micro heat exchangers, condensers, evaporators and boilers [4,9]; miniature refrigerators [10]; biomedical devices [8,11] cited by [12,13]; fuel cell technology [14,15,16], etc.

The above referenced equipment contains mini, micro or nano channels that can configure mini or micro heat exchangers. The microchannels can be circular, rectangular or have a different shape [3,13,17].

The need to use flow sections that are increasingly smaller for the heat exchange convective effect is based on the assertions of Kandlikar, Aubert et al. [3,8,13]. Since 1997, Phillips, R.J. et al. [18] indicated that for surfaces reaching a maximum of 120 °C, micro-channel heat sinks can dissipate heat loads as high as 1000 W/cm^2^. Such values must be attained in high performance cooling systems.

The present paper aims to analyze how the R134a coolant reacts in the rectangular microchannels of the vaporizer after an isenthalpic Joule-Thomson lamination process. Research in the field indicates that applying classic calculus methods to determine heat exchange in micro and nano channels is no longer a viable option. This can be explained by the fact that the size of the microchannel(s) is so small that it only gets close in scale to a couple of molecule layers. Hence, with regards to such extremely small scales, in 2003 Kandlikar and Grande [3] asserted that a rarefaction effect of common gases at atmospheric pressure is generated. Experimental research conducted so far shows that the heat transfer coefficients are either larger [19] or smaller [20] than the predicted values. It was also determined that deviations from the predicted values are registered depending on the flow conditions. In order for water to flow through rectangular microchannels, Peng et al. [21], demonstrated that the Reynolds number can have values ranging between 200–700 whilst Xu et al. [22] aimed for channels with similar dimensions, with Reynolds number values ranging between 20–4000. Liu, Harms, Popescu et al. [23,24,25] showed that the Reynolds number values vary significantly depending on the microchannel dimensions, near limit conditions, type of coolant used and the adopted mathematical model. These discrepancies can have direct effects on the design of microchannels in cooling systems.

The aim of this study is to examine if the values predicted through mathematical modelling for rectangular microchannels are close to the experimental values when subjected to a non-stationary heat exchange. Temperature distribution was analyzed when the R134a coolant flowed through rectangular microchannels at different flow rates, and also when the vaporizer was heated. In order to determine how the R134a coolant performs in rectangular microchannels, an experimental setup was designed and constructed as further shown.

## 2. Experimental Setup

**Figure 1:**
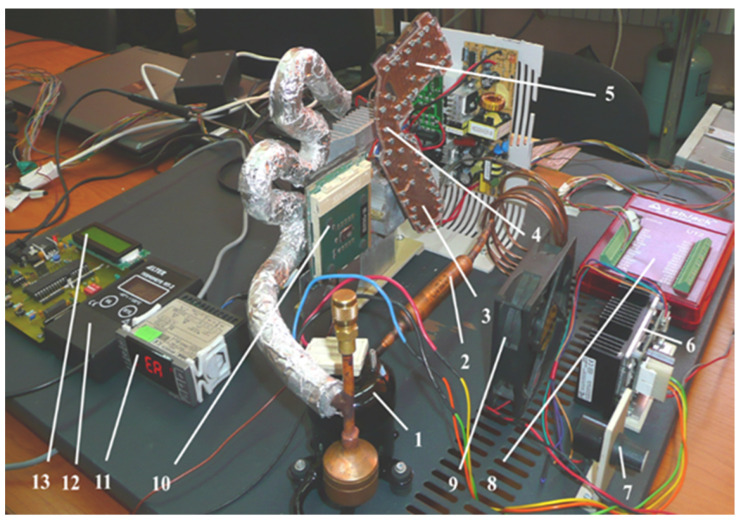
Image of the experimental setup: 1—ASPEN rotary compressor; 2—filter; 3—condenser; 4—lamination tube; 5—evaporator; 6—driver; 7—rotary switch used to adjust the speed; 8—LabJack U12 interface; 9—cooler; 10—CPU with a heat source located on the back side, not visible in the image; 11, 12, 13—thermometers.

Figure 1 illustrates an image of the experimental setup that allows monitoring of the temperature distribution of the R134a coolant flow through the components of the installation.

The test bench consists of an ASPEN Miniature Rotary Compressor 24 V, referenced as “1”, model 14-24-1101, preloaded with 21 cm^3^ of RL68 POE oil. The three-phase, direct current electric motor (up to 9.5 A) powered at 24 V, is equipped with a driver that ensures operation within 1800–7000 rev/min. When using the R134a coolant, at maximum revolutions, a heat dissipation power of 360 W is generated. From a design point of view, depending on the power supply parameters provided by the driver, the speed of the compressor can be varied accordingly. The mini cooling system’s compressor can provide different pressure variations whose values can be found in the technical documentation [26,27]. From compressor 1, the coolant flows through filter 2, and then through condenser 3 which is incorporated in the cooling system. Next, from the condenser, the R134a coolant flows through the lamination micro tube 4 and then through the vaporizer 5 which is incorporated in a cooling-lamination-heating plate. The elements positioned on the cooling plate have rectangular microchannels. The speed of compressor 1 can be varied through driver 6, using the rotary switch 7.

The LabJack interface, indicated by 8, allows determination of the temperature at various predefined measuring points in the system: at the compressor’s intake and exit points, as well as at the condenser’s and vaporizer’s entrance, middle and exit points. On the stand there is also a cooler 9 that serves the purpose of cooling the condenser. The vaporizer of the installation is in direct contact (through a thermal paste) with the CPU heat generating element, indicated by 10. The CPU was taken from a computer mainboard and was fitted, on its back side, with three DC powered electronic resistors, capable of reaching up to 110 °C. By raising the DC voltage of the power supply, any temperature ranging between the ambient value and maximum value can be obtained. It is required to know in advance the temperature to voltage ratio. Three other thermometers are also installed on the test bench. The one labeled 11 has the purpose of monitoring the temperature of the compressor and also of triggering a halt command for the compressor in case its temperature exceeds the maximum allowed temperature of 135 °C, per its manufacturing specifications. The thermometer labeled 12 is of a high precision type, serving the purpose of calibrating and measuring the local temperature at any point in the system, using a probe. Lastly, the thermometer labeled 13 allows for measurements of the ambient temperature. The installation is connected through a valve to a coolant tank. This allows monitoring of the volumes of coolant that are used by the mini-installation and to supplement the coolant when it registers losses.

If the drive motor speed is varied, different pressure values for the coolant can be obtained as it flows through the rectangular microchannels, which, in turn, determine flow-speed variations in the mini-refrigerating unit and, consequently, flow rate variations.

## 3. Characteristics of Rectangular Microchannels

**Figure 2:**
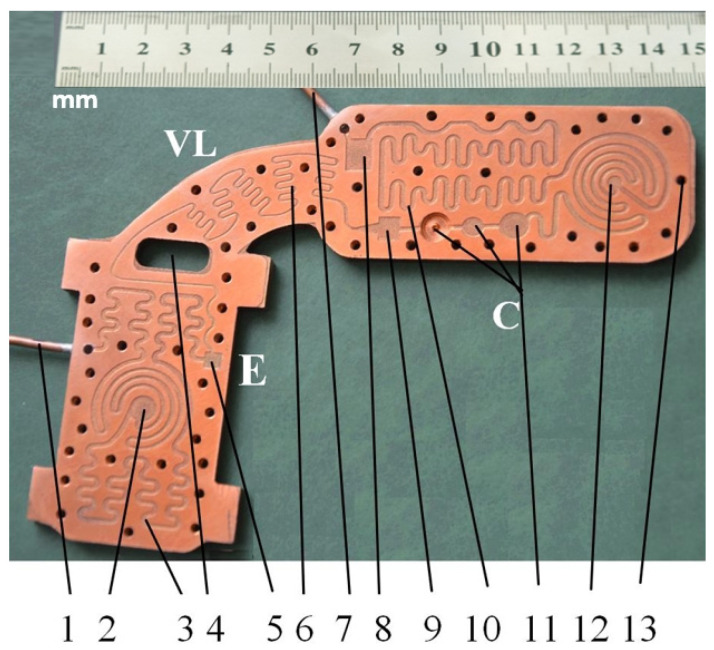
Condenser, lamination tube and the vaporizer of the mini refrigerating unit: 1—vaporizer drainage tube; 2—central area of the vaporizer; 3, 10—rectangular microchannels of the vaporizer, condenser, 4—shaft; 5, 8—mini filters; 6—rectangular microchannels in the lamination area; 7—compressor intake tube; 9—convergent nozzle; 11—mini liquid coolant tanks; 12—condenser cooling area; 13—mounting holes.

The cooling-lamination-heating plate was crafted out of copper with a 2.5 mm thickness and was designed for cooling the CPU (central processing unit). A technical problem that arose during the design stage was the high thermal conduction coefficient of this metal. Even if the channels are placed on the same board (which sustains an isobaric-isothermal condensation process, a lamination process Joule-Thomson, followed by an isobar-isothermal vaporization process), the conduction effect was avoided by the addition of channels and by creating thermal bridges on the back of the plates. A detailed image of the plate can be seen in Figure 2.

Tube 1 from Figure 2, connected with vaporizer E, removes the coolant that was vaporized by the heat generated from the CPU located in central area 2. In this area the vaporizer tends to balance the heat flow emanated by the CPU with the cold flow coming from the rectangular microchannels. The lowest temperature experimentally obtained at the vaporizer level was −22.3 °C. The coolant flows in the vaporizer through rectangular microchannels 3. Shaft 4 was designed in order to avoid conductive heat transfer between the vaporizer and the condenser. Due to the scale of the rectangular microchannels, and in order to avoid micro impurities or accidental vapor bubbles, a micro filter, 5, was mounted between the lamination valve 6 and microchannels 3. The coolant that results from the compressor’s output passes through tube 7 to micro filters 8 and 9 (intake, output) that are a part of condenser C. The coolant is directed to area 12 through the rectangular microchannels 10, while over area 12 a cold air stream passes as it comes from the mini-installation cooler. Because, at the delivery end of the condenser it is imperative that the coolant is fully converted to liquid, and also because to the low coolant flow through the installation, only a maximum quantity of 17 g of coolant was inserted, three cylindrical tanks of different volumes were fitted, indicated by 11. During the experiments, the coolant was observed in these tanks in its liquid stage. Throughout the heat exchange plate-outline, 13 threaded holes were drilled that allowed mounting of an acrylic transparent plate PMMA XT with a 2.5 mm thickness. This plate has a softening temperature of 115 °C, a specific heat capacity of 1.47 J/kg, a linear coefficient of expansion of 7 × 10^5^ K^−1^, heat conductivity of 0.18 W/mK, a maximum long-term temperature of 90 °C, a maximum short-term temperature of 110 °C and a refraction index of 1492 nD.

**Figure 3:**
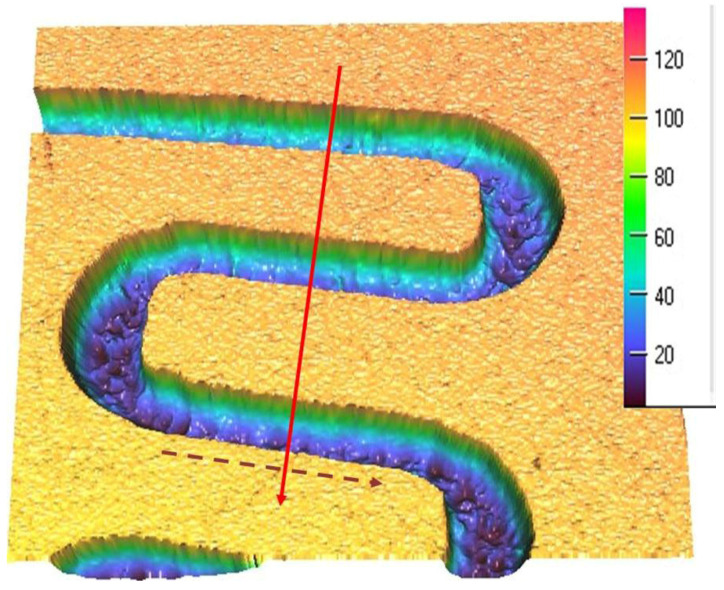
Rectangular microchannels from the condenser and the vaporizer, vertical scale in µm.

**Figure 4:**
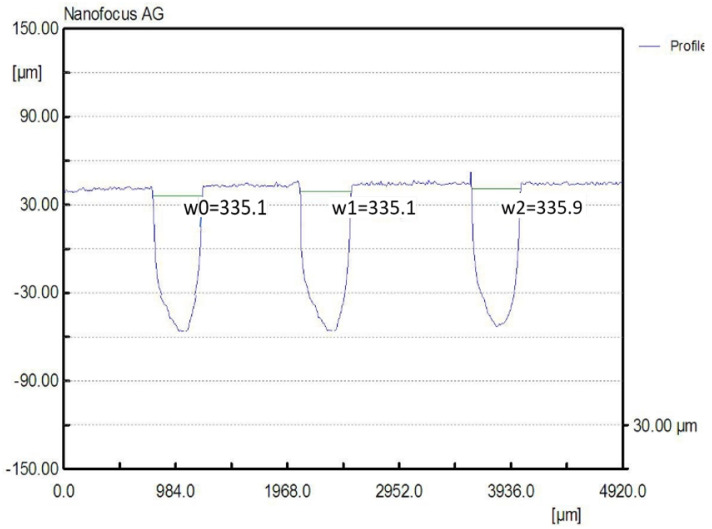
Transverse 2D profile and dimensions of the rectangular microchannels, obtained by laser profilometry.

The manufacturing of the rectangular microchannels was carried out using a laboratory method that consisted of multiple stages based on the photochemical imprinting of the material, followed by acid submersion. In order to compute the calculus and to determine the size of the mini-refrigerating unit it is important to know the scale of the rectangular microchannels and the roughness degree that resulted from their processing. The measured values can be used to determine any pressure decrease, the volume of the area through which the coolant flows, and the flow speed, as well as to study the influence of the roughness degree over the flow rate. For this purpose, the surface of the rectangular microchannels was mapped by the aid of laser profilometry. Typical measurement results obtained from the scanning process with regards to the main component parts of the mini refrigerating unit are further presented. Figure 3 shows a 3D representation of the rectangular microchannels that are identical for both condenser and vaporizer, and Figure 4 shows their 2D profile and dimensions.

For each scanned surface, values for the height and width of the rectangular microchannels were obtained in µm, as well as the inclination of the walls, compared to the vertical axis, and the surface roughness. Figure 4 illustrates an example of a transverse profile, highlighting the depth and width of the microchannels. The 2D profile shown in Figure 4 corresponds to the arrow shown in Figure 3. Based on the obtained results, no significant variations were found between the widths of different channels, with the difference amounting to maximum 0.8 µm in width and, respectively, 0.9 µm in depth. The shape of the channels at the base of the microchannels is shown to be different from a rectangular shape due to the limitations of the scanning equipment and image vertical scaling being similar, however, to the ones described in similar research.

**Figure 5:**
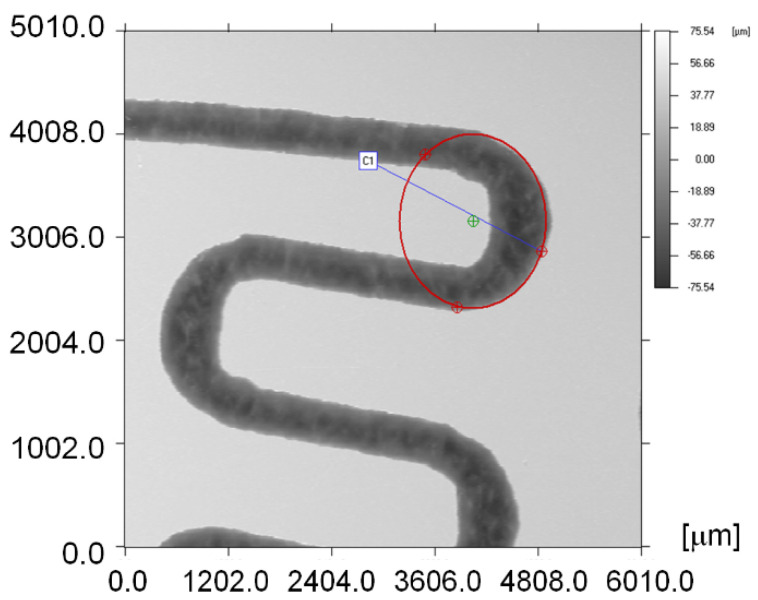
Measuring the curvature radius of the rectangular microchannels.

Figure 5 illustrates how the curvature radii of the rectangular microchannels were measured and in Table 1 a sample of such measurement results is presented.

In Table 1, X and Y represent the coordinates of the circle center by reporting to the initial reference point set for measuring. r is the circle radius and l is the length of the circle center position-vector versus the initial reference point. Values for circle C1 correspond to the exterior of the rectangular microchannel curvature and the values for circle C2 correspond to the interior of the rectangular microchannel.

**Figure 6:**
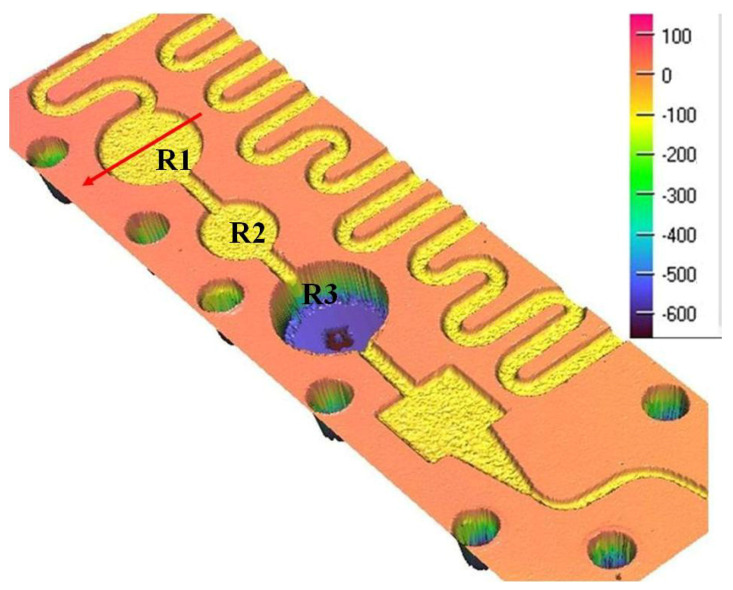
Additional coolant tank area and convergent nozzle, vertical scale in µm.

**Figure 7:**
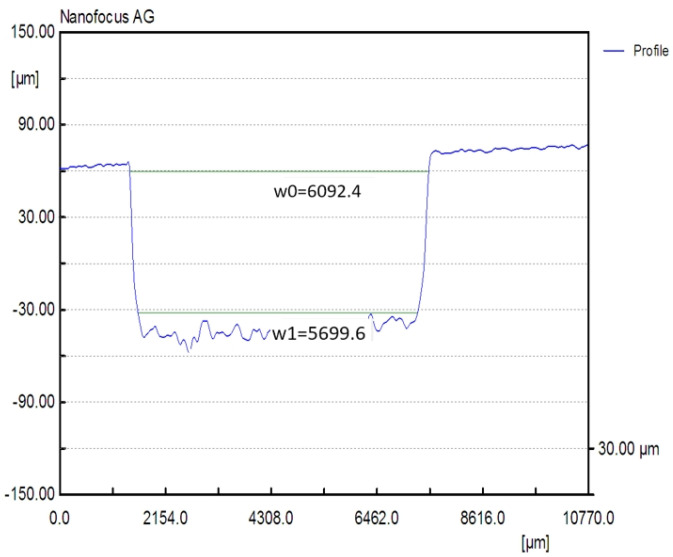
2D Transverse profile and dimensions of R1 coolant tank.

An example of the surface microtopography can be observed in Figure 6, including the additional R134a coolant tanks, as well as the convergent nozzle that precedes the lamination channel. Figure 7 shows a transverse profile and dimensions for the R1 tank.

The measurements computed in the laboratory, together with the ones presented in Figure 7, indicate the radius of the tanks r_R1,R3_ = 3046.2 µm, r_R2_ = 2311.4 µm and an average depth of h_R1,R2_ = 98.7 μm, while h_R3_ = 703 µm, based on other measurements.

**Figure 8:**
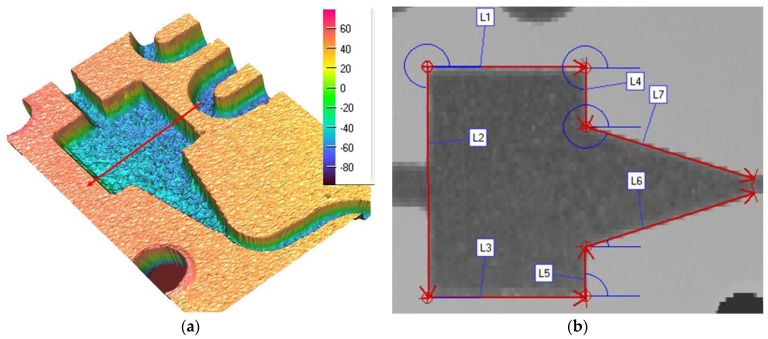
Convergent lamination nozzle area: (**a**) 3D representation, (**b**) measured regions.

Figure 8a shows a scanned image of the convergent nozzle that ensures the coolant flow from tanks R1–R3 to the lamination microchannel (scale is in μm), and Figure 8b presents all the areas where the measurements mentioned in Table 2 were carried out.

The convergent nozzle region dimensions shown in Table 2 are relevant when computing the parameters at the intake end of the lamination tube that ensures the Joule Thomson effect is achieved.

**Figure 9:**
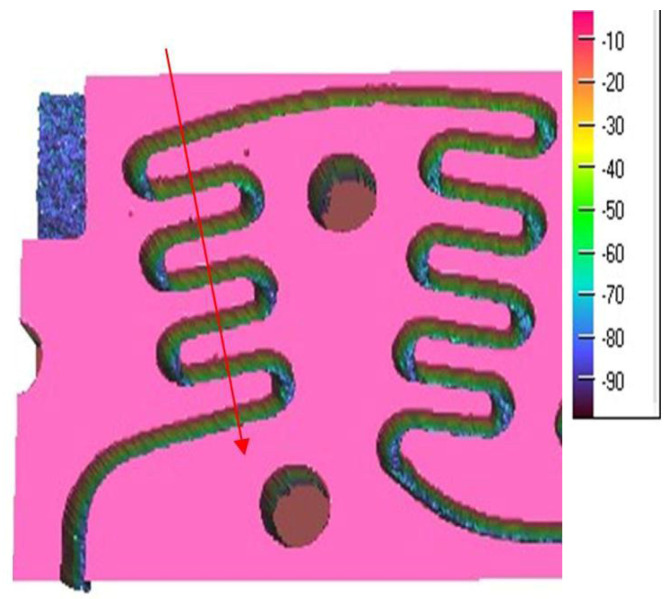
Rectangular microchannels used for laminating the HFC-R134a coolant, scale in µm.

**Figure 10:**
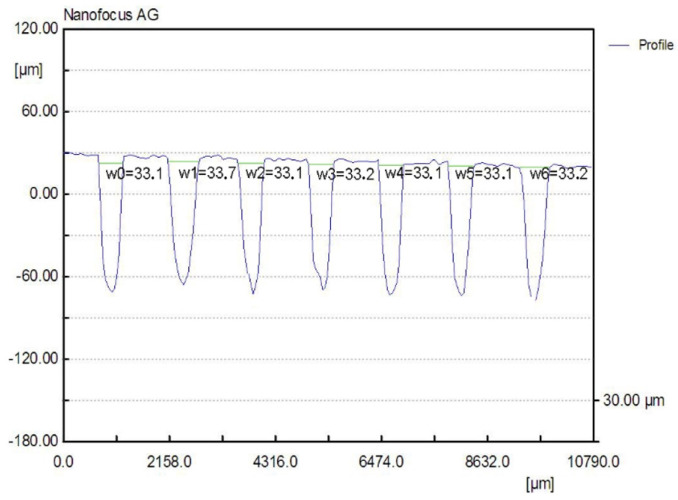
2D profile and dimensions of rectangular microchannels used for coolant lamination.

Figure 9 illustrates the direction used to generate the profile shown in Figure 10 from which the rectangular microchannels used to laminate the R134a coolant were measured. It is immediately obvious that these microchannels are much smaller in width compared with the ones from the condenser and the vaporizer. Due to technological reasons, the height of the rectangular microchannels is the same at the condenser, the lamination tube and the vaporizer, throughout the entire flow path of the coolant.

## 4. Temperature Variation of R134a Refrigerant during Vaporization in Rectangular Microchannels in a Transient Convective Regime

The aim of this research was to determine how the temperature variation of R134a coolant varies in rectangular microchannels. When coolant enters the vaporizer in a liquid state from the lamination capillary tube, at a certain well-determined distance, a liquid cone forms in the microchannel, inside which coolant vapors appear. This is followed by a portion of the microchannel in which the mixture is biphasic. The working fluid from the lamination capillary tube is discharged into the rectangular microchannels (with a much larger cross-section) which leads to negative temperatures. During the passage through the rectangular microchannels of the vaporizer, the coolant starts to boil, so that is in a vapor state at the outlet. It is necessary for the coolant to be in this state at the outlet of the vaporizer for it to be sucked in by the rotary compressor. In this process, transient convection heat transfer takes place.

**Figure 11:**
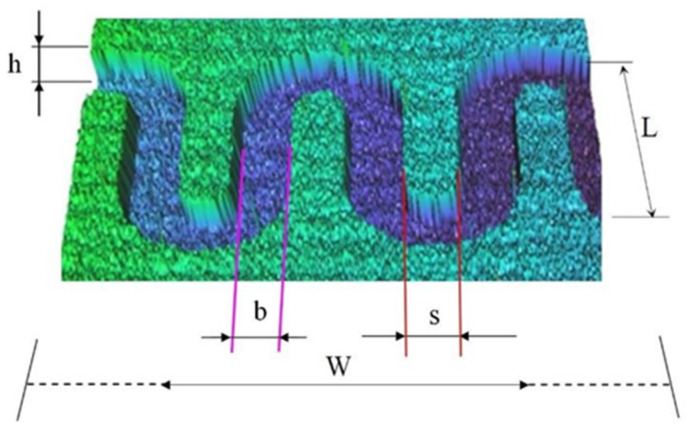
Micro-channel parameters considered for the analytical model.

Figure 11 shows a sequence of rectangular micro-channels belonging to a mini heat exchanger and subjected to a heat flow in the lower part in the case of the vaporizer.

For the calculations, it was considered that the R134a coolant had constant physical properties for a transient-state heat transfer, forced laminar flow regime. It was also considered that the flow was incompressible. It can be appreciated that the natural convection of heat was negligible. To simplify the calculations, it was assumed that the inlet temperature distribution was uniform and the temperature at the channel wall was prescribed and uniform.

For heat transfer, rectangular microchannels with mean width b = 335.36 μm, mean height h = 98.7 μm, mean distance between channels s = 378.9 μm and total length L1 = 8010 μm were considered.

The experimental setup, described above, allows the speed of the ASPEN rotary compressor model 14-24-1101 [26,27] to be changed between 1800–7000 rpm by means of the motor control driver at voltages between 1.13–4.94 V. It is necessary to consider in the calculations that changing the speed of the compressor also changes the flow rate of coolant through the rectangular microchannels. Applying the calculation model in [13], for the mini heat exchanger with efficiency η_f_ = 0.688, through which the R134a refrigerant flows the pressure drop, Reynolds number, fluid temperature at the inlet and outlet of the rectangular microchannels can be determined.

For the R134a refrigerant, the literature [28] recommends the values given in Table 3.

As the pressure in the installation changes depending on the speed of the ASPEN rotary compressor, there may be different values specific to the refrigerant properties. An example shown in Table 4 contains the values for the gaseous state of R134a refrigerant, for a pressure p_R134a_ = 2 × 10^5^ Pa, and a Temperature T_R134a_ = 22 °C, as given in the literature [29].

For the gaseous state of R134a coolant, the specific constant ℜ_R134a_ can be determined with Equation (1):

(1) $${ℜ}_{R134a}=\frac{p_{R134a}\frac{1}{\rho_{R134a}}}{Z_{cR134a}T_{R134a}},$$

where: p_R134a_—coolant pressure in rectangular microchannels [Pa]; ρ_R134a_—R134a density [kg/m^3^]; Z_cR134a_—coefficient of compressibility (dimensionless); T_R134a_—coolant temperature in rectangular microchannels [K].

For the calculation of the mean free path of coolant molecules R134a through rectangular microchannels, there are, according to Stéphane Colin [13], several models developed. For the adopted Variable Soft Spheres model, denoted VSS, the coefficient in the mean free path expression, k_mfp_ (dimensionless), is expressed with the relation:

(2) $$k_{mpf}=\frac{4\alpha_{VSS}\left(7-2\omega \right)\left(5-2\omega \right)}{5\left(\alpha_{VSS}+1\right)\left(\alpha_{VSS}+2\right)\sqrt{2\pi }},$$

where $\alpha_{VSS}$ is a characteristic coefficient for the VSS molecular model, for which a value of $\alpha_{VSS}=0.722$ (dimensionless) was adopted. The temperature exponent of the viscosity coefficient, ω (dimensionless) is:

(3) $$\omega =\frac{\eta +3}{2\left(\eta -1\right)}.$$

For the exponent in the inverse power law model, denoted by η, a value of η = 13 (dimensionless) was adopted, according to the values given by Lennard-Jones [7].

The mean free path λ, was determined as:

(4) $$\lambda =\frac{k_{mpf}\mu_{R134a}}{\rho_{R134a}\sqrt{{ℜ}_{134a}T_{R134a}}}.$$

The dynamic viscosity of the gaseous state R134a coolant was noted with μ_R134a_, and its value was taken from Table 4.

If the width of the rectangular microchannel b_ch_ = 335.36 μm and its height is h_ch_ = 98.7 μm the equivalent hydraulic diameter D_h_ can be calculated with the relation:

(5) $$D_{h}=\frac{4\left(b_{ch}h_{ch}\right)}{\left(b_{ch}+h_{ch}\right)}.$$

Calculations showed that λ = 8.063 × 10^−3^ μm and D_h_ = 305.03 μm.

For the VSS model, the Knudsen and Reynolds numbers are determined with the relations:

(6) KnVSS=λDh,Re=U(Y)DhνR134a.

The value obtained for K_nVSS_ = 2.643 × 10^−5^ indicates a continuum flow, according to the classification made by Stéphane Colin [13], with classical no-slip boundary conditions. It can be considered that the flow is accurately modelled by the compressible Navier-Stokes equations.

The developed velocity profile, U(Y), found by (Mikhailov and Cotta, 2004) [7] on the flow direction y, will thus be:

(7) $$U\left(Y\right)=\frac{6Kn_{VSS}\beta_{v}+3\frac{\left(1-Y^{2}\right)}{2}}{1+6Kn_{VSS}\beta_{v}},\;Y=\frac{y}{L_{1}}.$$

The dimensionless coefficient *βν* is obtained from:

(8) $$\beta_{v}=\frac{\left(2-\alpha_{m}\right)}{\alpha_{m}}.$$

In Equation (8), the dimensionless α_m_ coefficient represents the Eigenvalues for the velocity distribution in a rectangular microchannel.

**Figure 12:**
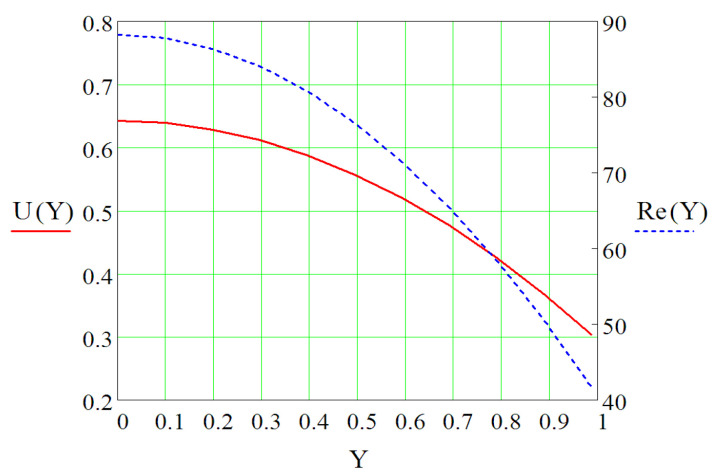
Velocity distribution U(Y) and Reynolds number Re(Y) for refrigerant circulation in rectangular microchannels as a function of path travelled (dimensionless values).

If it is considered that the total length of the vaporizer plate in which the rectangular microchannels are embedded is L_1_, from the calculations we obtained the representation in Figure 12 for the developed velocity profile is U(Y) and the Reynolds number, Re(Y). The figure shows how the velocity of Freon R134a along the rectangular microchannels in the vaporizer changes with respect to the dimensionless size Y.

Analyzing in Figure 12 the dimensionless flow velocity of the R134a coolant through the rectangular microchannels, there is a rather large drop of 53.81%, from the inlet value U(Y) = 0.695 to U(Y) = 0.374, which corresponds to the flow through the entire vaporizer duct. The shape of the velocity curve is similar to those presented by Stéphane Colin and Aubert [8,13]. The input/output value ratio determined in the present work of value Ui/Uo = 2.178, differs slightly from that determined by Aubert W*i/W*o = 2.25, respectively, Colin of value U*i/U*o = 2.66. According to the representation in Figure 12, the Reynolds number reaches a maximum of 88.258 and a minimum of 41.723, values that show, according to the classification made by Stéphane Colin [13] that the flow regime is laminar.

For dimensionless velocities of coolant in microchannels, considering ψ(Y) as the Eigen functions of the following Sturm-Liouville problem [7], we can write in simplified form, Equation (9):

(9) $$\frac{d^{2}\Psi_{i}\left(Y\right)}{dY^{2}}+\mu_{i}^{2}U\left(Y\right)\Psi_{i}\left(Y\right)=0,$$

where Y will have values between 0 and 1 and the original eigenvalues are obtained from:

$$\mu_{i}=\sqrt{\frac{2}{3}\left(1+6Kn_{VSS}\right)\nu_{i}},\;i=1,\;2,\;3\ldots .$$

The results are obtained by solving Equation (9) in terms of the confluent hyper geometric function, also known as the Kummer function _1_F_1_ [a; b; z]:

(10) $${}_{1}F_{1}\left(a;b;z\right)=\sum_{i=0}^{\infty }\frac{{\left(a\right)}_{i}z^{i}}{{\left(b\right)}_{i}i!},$$

where:

(11) $$a_{\left(i\right)}=\frac{1-\nu_{i}\left(1+Kn_{VSS}\beta_{v}\right)}{4},$$

*b_(i)_ = 1/2* for the considered case, and *z* will be:

$$z=\nu_{i}^{2},$$

This leads to:

(12) $$\Psi_{i}\left(Y\right)=\sum_{i=0}^{\infty }\frac{{\left(a\right)}_{i}z^{i}}{{\left(b\right)}_{i}i!}e^{\nu_{i}\frac{Y^{2}}{2}}.$$

The deduced Equation (12) satisfies Equation (9) and the conditions:

(13) $${\frac{d\Psi_{i}\left(Y\right)}{dY}|}_{Y=0}=0,\;Kn\beta_{v}\beta {\frac{d\Psi_{i}\left(Y\right)}{dY}|}_{Y=1}=-\frac{1}{2}\Psi_{i}\left(1\right).$$

The *β* coefficient is determined with the relation:

(14) $$\beta =\frac{\beta_{t}}{\beta_{v}},\beta_{t}=\frac{\frac{2-\alpha_{i}}{\alpha_{i}}\frac{2\chi }{\chi +1}}{\mathrm{Pr}},$$

where *α_t_* is the thermal accommodation coefficient, *χ* the adiabatic exponent and *Pr* is the Prandtl number. 

In this case the Eigen condition is set:

(15) $$\left\{2Kn\beta_{v}\beta_{1}F_{1}\left[\frac{5}{4}-T_{1},\frac{3}{2},\nu_{i}\right]\nu_{i}\left(1-4T_{i}\right){+}_{1}F_{1}\left[\frac{1}{4}-T_{1},\frac{1}{2},\nu_{i}\right]\left(1-2Kn\beta_{v}\beta \nu_{i}\right)\right\}e^{\frac{\nu_{i}}{2}}=0,$$

where: $T_{1}=\frac{\nu_{i}\left(1+4Kn\beta_{v}\right)}{4}$.

The equation is a function of two parameters Knβ_ν_ and β. From Equation (15), the eigenvalues, ν_i_, and the original eigenvalues, µ_i_ can be determined. 

The normalization integral coefficient of the function is:

(16) $$N_{i}=\int_{0}^{1}U\left(Y\right){\left(\Psi_{i}\left(Y\right)\right)}^{2}dY.$$

The normalized form of the Eigen function is:

(17) $${\tilde{\Psi }}_{i}\left(Y\right)=\frac{\Psi_{i}\left(Y\right)}{\sqrt{N_{i}}}.$$

The transform-inverse pair is:

(18) $$\theta_{im}\left(Z,\tau \right)=\int_{0}^{1}U\left(Y\right)\tilde{\Psi_{i}}\left(Y\right)\theta \left(Y,Z,\tau \right)dY,\tau =\frac{\alpha t}{L_{1}^{2}},Z=\frac{1}{Pe}\frac{z}{L_{1}},$$

where τ is the dimensionless time, equal to the product of α (α = k/ρc_p_ where k is the thermal conductivity divided by the product of density and specific heat at constant pressure) and a characteristic time, divided by the quadratic distance from the middle of the body through which the heat passes to the surface, *Pe* is the Peclet number. The inverse of the function is:

(19) $$\theta \left(Z,Y,\tau \right)=\sum \tilde{\Psi_{i}}\left(Y\right)\theta_{im}\left(Z,\tau \right),$$

The value of the dimensionless mean temperature drops as a function of τ is:

(20) $$\theta_{av}\left(Z,\tau \right)=\frac{\int_{0}^{1}\left[\frac{6Kn_{VSS}\beta_{v}+3\frac{1-Y^{2}}{2}}{1+6Kn_{VSS}\beta_{v}}\sum_{i=1}^{\infty }\tilde{\Psi_{i}}\left(Y\right)\theta_{im}\left(Z,\tau \right)\right]dY}{\int_{0}^{1}\frac{6Kn_{VSS}\beta_{v}+3\frac{1-Y^{2}}{2}}{1+6Kn_{VSS}\beta_{v}}dY},$$

**Figure 13:**
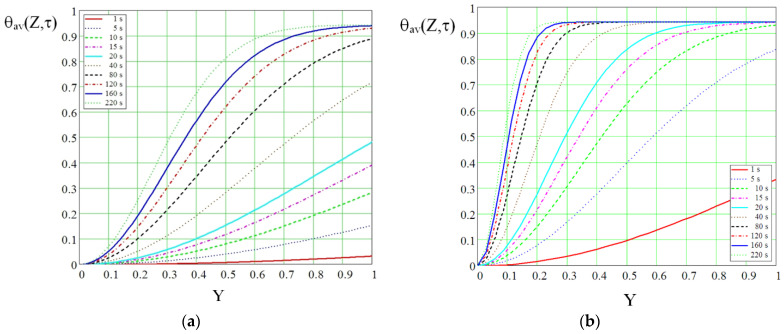
Evolution of the mean dimensionless temperature over time: (**a**) without heat source at the vaporizer, (**b**) with heat source at the vaporizer.

Figure 13a,b, illustrate the evolution of the dimensionless mean temperature obtained by implementing the above described equations in a Mathcad code [30]. The presence and absence of a heat source at the vaporizer are considered.

**Figure 14:**
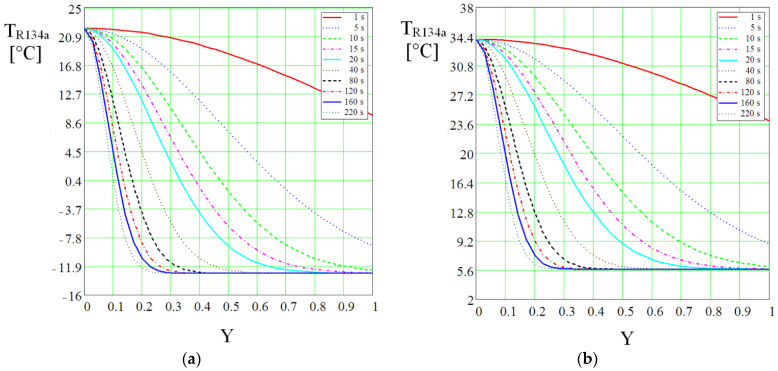
Temperature change in non-stationary mode for R134a coolant along the rectangular microchannels of the vaporizer at compressor speed of 4400 rpm: (**a**) without hot source, (**b**) with hot source at T = 34 °C.

In order to determine the temperature drop along rectangular microchannels, taking into account velocity and time, the following equation is used:

(21) $$T_{R134a}=T_{in}-\theta_{av}\left(Z,\tau \right)\left(T_{in}-T_{ou}\right),$$

where *T_in_*, *T_ou_* are the temperatures of R134a coolant at the inlet and outlet of the vaporizer’s rectangular microchannels. Figure 14 shows the temperature evolution in the rectangular microchannels belonging to the vaporizer as a function of time and the dimensionless value Y.

If it is assumed that the vaporizer is not heated by the hot source, according to the calculations and Figure 14a, it is found that the temperature decreases in the center of the vaporizer from an initial value of +21.8 °C to a minimum value of −12.3 °C. If the non-stationary behavior is analyzed in the same figure, it can be observed that after 8 s the coolant temperature drops to −9.8 °C. This confirms that the Joule-Thompson effect followed by expansion in the vaporizer takes place rapidly in the rectangular microchannels, but a strong rarefaction effect also occurs. After 60 s of flowing through the rectangular microchannels, the cooling regime stabilizes, and the curves have the same shape and close values.

For the case of heating the center of the vaporizer from the calculations and Figure 14b, the refrigerating agent which initially has a temperature of +34.1 °C cools down in the center of the vaporizer to a minimum value of +5.6 °C. Concerning the non-stationary behavior, the calculations show that after 5 s, the coolant temperature in the rectangular microchannels decreases to +9 °C.

## 5. Experimental Determinations of R134a Coolant Temperature Evolution during Mechanical Compression in Rectangular Microchannels

In order to be able to make a comparative analysis of the data obtained from the calculations with experimental results, it is considered appropriate to present below the values determined on the mini refrigeration unit with rectangular microchannels mechanically compressing the R134a coolant.

Temperature was measured experimentally at various points of interest in the installation using a LabJack U12 digital-analogue interface to which eight LM15 temperature sensors were connected. The LabVIEW environment was used to read the data. The temperature sensors provide values for: CI—compressor inlet; CD—compressor discharge; IC—condenser inlet; CC—condenser central area; OC—condenser outlet; IV—vaporizer inlet; CV—vaporizer central area; and OV—vaporizer outlet.

The temperature distribution of R134a coolant in rectangular microchannels at different points of the plant was obtained for the following situations:

- the rotational speed of the ASPEN rotary compressor is changed, in which case according to the technical documentation [26] and the research carried out [27] different flow rates are obtained in the microchannels of the mini refrigeration plant;
- the same compressor speed is maintained, but the heat generated by a centrally located hot source behind the evaporator is modified.

Changing the speed of the ASPEN rotary compressor is easily obtained in practice, using a potentiometer attached to the control driver.

The temperature change in the central area of the evaporator was achieved with the help of three electrical resistors located inside an AMD CPU. These were DC powered at different voltages using a voltage source. The electrical resistors simulate the heart of the CPU and can reach a maximum of 110 °C, their temperature being measured with an electronic thermometer.

Experimental results for the two cases are presented in the following sections.

### 5.1. R134a Coolant Temperature Evolution at Variable Flow Rate through Rectangular Microchannels

**Figure 15:**
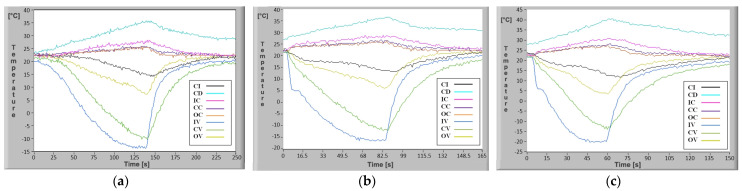
Temperature evolution in rectangular microchannels of the mini refrigeration unit for ASPEN rotary compressor different speeds: (**a**) 1800 rpm, **(b**) 4400 rpm, (**c**) 7000 rpm.

Since the experimental determinations were carried out under real operating conditions, it is interesting to see how the temperature evolves in the rectangular microchannels and in the various elements of the mini refrigeration unit. From the many possible speeds ranging from 1800–7000 rpm, it was considered that for the interpretation of the evolution of the parameters in the mini system, the results obtained for the minimum, the average and the maximum speed were sufficient. In Figure 15a–c, the results of the experimental determinations can be visualized as time-dependent plots.

The obtained results allow analysis of how the system temperature evolves as a function of increasing coolant flow in the rectangular microchannels. It should be noted that since the ASPEN rotary compressor and the drive motor are encapsulated, the speeds are assessed based on technical data provided by the supplier [26,27]. The analysis of the parameters obtained is the basis for determining the optimum operating speed.

### 5.2. R134a Coolant Temperature Evolution at Constant Flow Rate through Rectangular Microchannels and Central Heating of the Vaporizer

**Figure 16:**
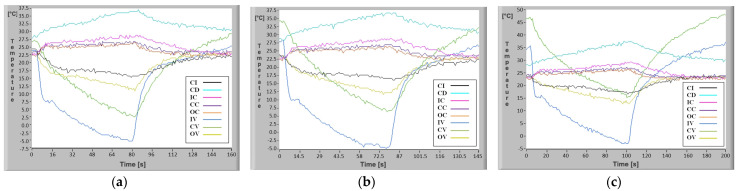
Temperature evolution in rectangular microchannels of the mini refrigeration unit for electrical resistors in the central area of the evaporator heated to (**a**) 26 °C, (**b**) 34 °C, and (**c**) 47 °C.

As stated, the mini refrigeration system was tested in the case of central heating of the vaporizer by means of the processor denoted by 10 in Figure 1, which had three DC powered electrical resistors applied at various voltages. Basically, three heating presets were used and the obtained measurement results are shown in Figure 16a–c.

It was experimentally found that significant differences occur between the temperature of the electrical resistors (50°, 70°, 90 °C) and the values reached at the CPU-vaporizer interface (26°, 34°, 47 °C). This can be explained by the existence of inner layers of the CPU, surface roughness, etc.

The experimental results shown in Figure 15a–c and Figure 16a–c illustrate the temperature variation in a transient regime in any point of the mini refrigeration unit with rectangular microchannels and highlight the temperature decrease speed at the vaporizer level, depending on the compressor speed.

## 6. Discussion

The present paper describes temperature distribution in the rectangular microchannels of a mini refrigeration unit using mechanical compression of R134a coolant, depending on operating conditions. It was experimentally found that a minimum temperature of −22.3 °C is reached in the vaporizer. The dimensions of the vaporizer are only 47.2 mm × 18.7 mm.

To perform the calculations it is necessary to know in detail both the dimensions of the rectangular microchannels and the values of the roughness of the flow channels. In usual flow channels the roughness influences the flow parameters only to a small extent, but in the case of micro and nano channels, the thickness of the liquid film or vapor jet can have values sensitively close to those of the surface asperities. Rectangular microchannel sizes differ depending on the operating area. Rectangular microchannels, as shown in Figure 3, Figure 4 and Figure 5, have values that are close to each other at the condenser and evaporator, with mean widths of 335.36 μm, mean depths of 98.7 μm and an average radius of channel curvature of 659.63 μm. For the same mean depth, in the laminating region, according to Figure 9 and Figure 10, the rectangular microchannel width is only 33.21 μm. This narrowing of the flow section is mandatory to obtain the Joule-Thompson effect. Since microchannels are used, it was considered useful in the design to use three tanks for storing the freon in the liquefied state. Tanks R1 and R3 in Figure 6 have a radius of 3046.2 μm while the radius of reservoir R2 is 2311.4 μm. At an average depth of 98.7 μm for R1 and R2, respectively, 703 μm for R3, a liquid freon reservoir of 25.027 mm^3^ is obtained, which ensures the avoidance of its gaseous penetration into the rolling tube.

Starting from the dimensionless mean values of the coolant temperature at transient flow through the rectangular microchannels determined by calculus for the vaporizer, the temperature change as a function of time can be analyzed.

Calculations were performed for two cases, as further discussed. 

The average experimental values determined for the mini refrigerating unit’s vaporizer are shown in Table 5.

Experimentally, it was analyzed how the R134a coolant behaves while traveling through the rectangular microchannels in three situations: changing the compressor speed (implicitly the flow rate), and maintaining the compressor speed at 4400 rpm without and with heating of the vaporizer central region.

For the case of changing the flow rate when the ASPEN rotary compressor speed reaches 1800, 4400 and 7000 rpm without heating the vaporizer, as shown in Figure 15a–c, the minimum temperatures reached at the vaporizer vary in quite a wide range, from −13.3 °C to −22.3 °C. The time in which the mentioned negative values are reached ranges from 120 s, corresponding to the minimum speed, up to 48 s, which corresponds to a compressor speed of 7000 rpm. It is immediately clear that the duration of the cooling process is 2.5 times shorter at maximum speed than at minimum speed. Experimentally, however, it was found that although the minimum temperatures are reached much faster at maximum speed, the compressor also heats up much faster. Analysis of the behavior of a cooling agent in rectangular microchannels with a changing flow rate is necessary because of the strong rarefaction effect due to extremely small flow cross-sections. In operation, this strongly affects the temperature and noise of the compressor which changes significantly with speed. Based on the minimum temperature value required to be achieved with the mini refrigeration unit, the noise produced and the compressor’s maximum bearable heating temperature, it was found that operation is optimal at 4400 rpm.

In the case where the vaporizer does not receive additional heat, at 4400 rpm the corresponding experimental results shown in Figure 15b (CV curve), indicate the temperature variation in the central region of the vaporizer independent of time. After 8 s there is a sudden drop in temperature from 21.8 °C to +12 °C, not reaching the −9.8 °C determined theoretically. This can be explained by the inertia of the temperature sensor and the fact that it was not placed inside the rectangular microchannel, but close to it. However, after 69 s when the system is switched off (the stabilized minimum temperature regime has been reached) the temperature value in the experimental case is −11.7 °C compared to the theoretical case when it is −12.3 °C. The temperature difference of 0.5 °C between the results of the mathematical calculations and the experimental values can be explained by losses in the mini refrigeration unit and measurement errors.

If the center of the vaporizer is heated, the experimental determinations shown in Figure 16b (CV curve) indicate a sudden drop in temperature after 8 s of operation from 34.1 °C to 27.1 °C. It is also noted that the theoretical value of 7.8 °C is not reached. After the 72.5 s, when the unit stops at the minimum stabilized temperature regime, the temperature value in the theoretical case is 5.6 °C and in the experimental case 6.3 °C. As in the previous case there is a small difference in temperature for the two cases of 0.7 °C but also in this case it can be appreciated that there is a very good correlation between the results of the mathematical calculations and the experiment.

The mini-installation with rectangular microchannels mechanically compressing R134a refrigerant using an ASPEN rotary compressor, although using only 17 g of fluid, provides a maximum refrigerating capacity of 1230 Btuh. In the refrigerating mini-installation’s vaporizer, of only 47.2 mm × 18.7 mm, a temperature of −22.3 °C was obtained experimentally compared to the theoretical value of −24 °C. It was found that although the flow cross-section of the rectangular microchannels has a strong rarefaction effect it is possible to functionally obtain mini refrigeration units for cooling in various fields of application.

## 7. Conclusions

The mini refrigeration unit experimentally built and presented in this paper offers the novelty of implementing microchannels in all its regions. The dimensions of the rectangular microchannels shown in Figure 6, Figure 7, Figure 8 and Figure 9 were obtained by laser profilometry. The experimental measurements show how the rectangular microchannels were dimensioned for each region of the mini refrigerating unit so that a maximum specific cooling capacity was obtained. Another novelty element introduced by the presented experimental setup was that all the functional regions of the refrigerating unit were placed on a single plate with rectangular microchannels, as shown in Figure 2, which allows for a very compact unit.

Table 6 presents some comparative specifications of the above presented mini refrigerating unit with microchannels, compared to similar units found in the literature [31,32,33].

The convenient placement of eight temperature sensors and real time signal acquisition allowed plotting of the real temperature evolution in key points of the mini refrigerating unit, as shown in Figure 15 and Figure 16, which in turn allows study of the temperature-variation laws over time.

The mini refrigerating unit described in the present paper offers several advantages, starting with an important specific cooling capacity of 328.18 W/kg, due to the implementation of rectangular microchannels in the condenser, vaporizer and lamination regions. The data illustrated in Table 6 show that the use of rectangular microchannels that are only 33.21 µm wide (in average) significantly increases the specific cooling capacity (by 61.85% in the studied case) by reporting to other mini cooling units. This is achieved while the unit is very compact as its dimensions are 59.89% smaller than the highest performing similar unit. The obtained performances are also remarkable for the fact that the described mini refrigerating unit only uses 17 g of R134a coolant.

The described mini refrigerating unit equipped with rectangular microchannels was tested in a laboratory over the course of a year without intervening on its constructive elements. This shows that this set-up can be used to obtain very reliable cooling equipment that uses microchannels and only small amounts of cooling agent.

## 8. Patents

System with rectangular micro- and nano-channels for freon processor cooling.

- Patent Number(s): RO129915-A2
- Inventor(s): MIHAI I, OLARIU E
- Patent Assignee Name(s) and Code(s): UNIV SUCEAVA STEFAN CEL MARE (UYSU-Non-standard)
- Derwent Primary Accession Number: 2015-10385E

## Figures and Tables

**Table 1:** Rectangular microchannels measured curvature radii.

Circle	X [µm]	Y [µm]	r [µm]	l [µm]
C1	4052	3166	852.68	5143
C2	3814	3161	466.58	4953

**Table 2:** Dimensions of the convergent nozzle.

Line	L [µm]	dz [µm]	Angle [°]
L1	4554	−4.66	0.000
L2	5163	3.24	270.290
L3	4528	−0.76	0.000
L4	1345	−79.25	272.230
L5	1091	−90.33	90.000
L6	4965	18.42	14.081
L7	4976	19.39	346.645

**Table 3:** Properties of Coolant R134a in gaseous and liquid state at atmospheric pressure.

Gaseous Phase			Liquid Phase		
Property	Value	UM	Property	Value	UM
Gas density (1.013 × 10^5^ Pa)			Liquid density		
(at boiling point)	5.28	kg/m^3^	(1.013 × 10^5^ Pa and 25 °C)	246.6	kg/m^3^
(1.013 × 10^5^ Pa and 15 °C)	4.25	kg/m^3^			
Compressibility factor (Z)			Boiling point		
(1.013 bar and 15 °C)	1	-	(1.013 × 10^5^ Pa)	1206	K
Specific volume			Latent heat of vaporization	215.9	kJ/kg
(1.013 × 10^5^ Pa and 15 °C)	0.235	m^3^/kg	(1.013 bar at boiling point)		
Heat capacity at constant pressure (Cp)			Vapor pressure		kPa
(1.013 × 10^5^ Pa and 25 °C)	87.54	J/kgK	(at 5 °C)	350	
			(at 15 °C)	490	
			(at 20 °C)	570	
			(at 50 °C)	1320	
			Heat capacity at constant pressure (Cp)		
			(1.013 × 10^5^ Pa and 25 °C)	1441	J/kg⋅K

**Table 4:** Gaseous state R134a refrigerant properties.

Gaseous State R134a Coolant		
Property	Value	UM
Density	9.0613	kg/m^3^
Specific enthalpy	419.88	kJ/kg
Specific entropy	1.8547	kJ/kg K
Specific isobar heat capacity	0.87506	kJ/kg K
Isobar coefficient of thermal expansion	4.077	10^−3^ (1/K)
Heat conductance	13.5875	10^−3^ (W/mK)
Dynamic viscosity	11.885	10^−6^ (Pa s)
Kinematic viscosity	2.2199	10^−6^ (m^2^/s)
Thermal diffusivity	29.655	10^−7^ (m^2^/s)
Prandtl-number	0.765	-
Compressibility coefficient	0.95505	-

**Table 5:** Experimental parameters in the vaporizer region.

Heat Source	Initial Vaporizer Temperature	Temperature at Vaporizer Inlet (IV)	Temperature in Vaporizer Central Region (CV)	Temperature at Vaporizer Outlet (OV)	Time	Rotary Compressor Speed
Unit	°C	°C	°C	°C	s	rpm
	23	−13.3	−8.9	+7.5	120	1800
NO	23	−16.1	−12.3	+6.8	69	4400
	23	−22.3	−14.4	+4.2	48	7000
	26	−5.0	+3.2	+11.3	71.2	4400
YES	34	−3.2	+7.1	+12.5	72.5	4400
	47	−2.5	+17.0	+13.7	93.4	4400

**Table 6:** Mini refrigeration unit.

Model	Application	Power Source [V]	Cooling Capacity [W]	Specific Cooling Capacity [W/kg]	Condenser PCS	Dimension L × W × H [mm]
Proposed mini refrigerating unit with microchannels	PCC/MF/PR/ LSC/HBC	DC24	360	328.18	Microchannel	170 × 120 × 115
Purswave Qx36W, [31]	PCC/MF/PR/ LSC/HBC	DC24	365	122.48	Fin	470 × 180 × 160
HACF405DC12, [32]	MBP/LBP	DC12	405	135.00	Fin	250 × 160 × 170
HAC550DC24, [32]	MBP	DC24	550	157.14	Fin	250 × 160 × 260
HAF115DC24-L, [32]	LBP	DC24	115	32.85	Fin	320 × 110 × 200
HAF115DC24-V, [32]	LBP	DC24	115	32.85	Fin	190 × 160 × 200
FSCD019Z12, [33]	MC/MAC/MF/MCH	DC12	365	202.77	Microchannel	180 × 170 × 128
FSCD0325Z24, [33]	TCW/MWC/EV	DC24	550	183.33	Microchannel	250 × 160 × 170

where: PC, CPU, sever cooling—PCC; medical facilities—MF; portable refrigerator—PR; limited space cooling—LSC; human body cooling—HBC; mini-cooler—MC; medium back pressure—MBC; low back pressure—LBC; mini air conditioner—MAC; mini freezer—MF; mini chiller—MCH; tiny cold-water machine—TCW; micro water chiller—MWC; electric vehicle air conditioning—EV.

## Data Availability

Some or all data, models, or code generated or used during the study are available from the corresponding author by request.
